# Caribbean Diaspora Healthy Nutrition Outreach Project (CDHNOP): A Qualitative and Quantitative Approach to Caribbean Health

**DOI:** 10.5334/aogh.2657

**Published:** 2020-02-04

**Authors:** Farzanna S. Haffizulla, Anjali Ramoutar, Alyssa Eason, Patrick Hardigan

**Affiliations:** 1 Dr. Kiran C. Patel College of Allopathic Medicine, Nova Southeastern University, Davie, US

## Abstract

**Background:**

Obesity prevention and its associated co-morbidities such as diabetes require a multi-tiered, culturally sensitive, population-based approach. South Florida’s tri-county area is home to approximately 75% of Florida’s total Caribbean immigrant population. This project is the first Caribbean-focused intervention using the *Go-Slow-Whoa* or *GSW* format which designates whether a food or beverage should be chosen frequently (*Go* – green), less often (*Slow* – yellow), or rarely (*Whoa* – red) based on the content of nutrients, sodium, fat, and sugar.

**Specific Aims:**

1. To create and evaluate culturally appropriate nutrition materials for the Caribbean diaspora population in Broward County (i.e. tailor existing GSW evidence-based materials for this population). 2. To quantify which social determinants of health are most relevant to this population.

**Methods:**

Mixed methods were utilized in this study. The qualitative, exploratory arm consisted of semi-structured focus groups that included 38 subjects from five Caribbean countries most represented in South Florida: Jamaica, Haiti, Trinidad and Tobago, Cuba and Dominican Republic. The quantitative arm employed descriptive and inferential statistics to analyze social determinants of health (SDOH) obtained from a modified National Association of Community Health Centers’ PRAPARE survey. Intercept survey data was also collected from a convenience sample of 24 Caribbean immigrants in Broward County.

**Findings:**

Analysis revealed a lack of culturally appropriate foods and exercise examples in the current *GSW* materials. At 92% and 82% respectively, an overwhelming majority of our intercept surveys indicated that our revised, culturally appropriate materials were helpful in making positive food and beverage choices. Further study is required to determine which SDOH variables are relevant to this population.

**Conclusions:**

Health disparities and inequity in the healthy living education of our Caribbean subpopulation are best addressed using an inclusive research frame that captures the cultural essence and preferences of this understudied community.

## Introduction and Purpose

The Caribbean Diaspora Healthy Nutrition Outreach Project (CDHNOP) aims to improve the overall health and living experience of Caribbean immigrants and their families. The Caribbean diaspora population in the United States consists of individuals who are either born in the Caribbean or are of Caribbean descent and are currently living in the United States [1]. Despite the substantial proportion of Caribbean diaspora residing in the US, especially South Florida, there is a lack of representation in national data, further exacerbated by lack of research and intervention. Through our data collection and analysis, this longitudinal research is anticipated to identify key patterns in diet & nutrition, as well as social determinants of health. Furthermore, we plan to recognize the health risks that can be acted on through effective, culturally appropriate interventions. According to Zong and Batalova, in 2014, approximately 4 million immigrants from the Caribbean resided in the United States, accounting for 9 percent of the nation’s 42.4 million immigrants [1]. More than 90 percent of Caribbean immigrants came from five countries: Cuba, the Dominican Republic, Jamaica, Haiti, and Trinidad and Tobago. The article further states that Caribbean immigrants were heavily concentrated in Florida (40 percent), New York (28 percent), and to a lesser extent, New Jersey (8 percent), according to 2010–14 ACS data. According to the Migration Policy Institute, Florida has the largest population of Caribbean immigrants in the United States [1]. A total of 1.645 million Caribbean immigrants live in Florida, representing approximately 41% of the Caribbean immigrant population in the United States. South Florida’s tri-county area (Miami-Dade, Broward and Palm Beach counties) is home to approximately 75% of Florida’s total Caribbean immigrant population. In addition, there are an estimated 232,000 unauthorized Caribbean immigrants in the United States, with Florida being the state with the second largest population of unauthorized Caribbean immigrants [1]. Data from the Broward County Department of Health (DOH) show a 60% and 80% increase in the West Indian and Cuban populations respectively from 2000 to 2014 [2].

## Disease/Comorbidities

Diabetes is a predominant disease that affects the Caribbean diaspora. However, there is limited literature describing the disparities regarding the Caribbean population compared to other racial and ethnic populations. Other studies previously have suggested that both Africans and Caribbean diaspora experience higher odds of diabetes despite lower obesity rates than that of US-born blacks [3]. This indicates that risk profiles differ based on country of origin.

## Cultural Risk Factors

A secondary cross-sectional analysis of New York Africans and Caribbean diaspora examined the relationships between the two demographics. Caribbean immigrants experienced a higher prevalence in both diabetes and obesity than Africans. Although they had similar chances of developing diabetes as Africans, they were found to have a higher probability of developing obesity. Although obesity is a known risk factor in diabetes, obesity prevalence and odds did not always correlate with those of diabetes [4]. This reinforces the idea that there are cultural factors that may influence disease prevalence and odds. Gender may also be another factor influencing disease prevalence and odds. In both demographics, women were significantly more likely than men to experience obesity. Gender may be an even stronger predictive factor in diabetes because Caribbean women had a higher prevalence of obesity, and there was no difference in diabetes prevalence between Caribbean men and women. Further studies investigating the heterogeneity of risk profiles within these ethnic groups can optimize interventions to better diabetes morbidities and reduce adverse health outcomes [3]. A review of diabetes in Caribbean populations reported that overall, the prevalence of diabetes mellitus was higher in Afro-Caribbean populations when compared to Caucasian or other African populations. Within these ethnic groups, diabetes prevalence was seen to be increased in women when compared to men. Additionally, prevalence, morbidity, and mortality of diabetes were associated with lower SES [4,5].

## Cultural Influences

Societal and cultural norms significantly influence health behaviors and dietary intentions [5]. Cultural influences are an important factor in the ways that individuals adjust and maintain their eating behaviors. Bramble et al. found that indigenous traditions influenced the ways Afro-Caribbean and African American women approached food and preparation [5]. While the women interviewed in the study faced similar challenges, such as difficulty accessing fresh organic produce and exercise facilities, they also had several differences. Afro-Caribbean women stated they were more accustomed to daily walking and used to eating foods that were processed or refined. Interestingly, participants reported changes in their eating and exercise habits as a result of coming to the United States. In fact, it has been demonstrated among Latino immigrants that factors such as exposure to high portion meals, energy-dense meals, and mass media regarding food and sedentary lifestyles were perceived as being significant factors in leading to obesity and the overall acculturation process [5].

## Health Disparity Assessments

According to July 2015 data from the Broward Regional Health Planning Council’s Broward County Health Assessment Executive Report, a total of 2 in 3 Broward County adults (66.6%) are overweight, and another 24.3% are obese. Obesity and related diseases such as diabetes (13.8%), heart disease (6.6%), hypertension (36.5%), and stroke (2.5%) continue to rise in Broward County [6]. According to the Florida Department of Health in Broward County’s June 2016, Community Health Needs Assessment, the percentage of Broward County adults who are obese is higher than that of the peer county average. There is a disparity in the percent of black Broward County adults who are obese [2]. This percentage does not meet the Healthy People goal of 30.5% by 2020. Further data analysis from the County Health Assessment (CHA) reveals 66.6% of residents are overweight, higher than the national average and not meeting the Healthy People 2020 benchmark goal of 33.9% for normal weight [6]. According to the 2016 American Community Survey by the U.S. Census Bureau, there were over 277,000 residents from the Caribbean/West Indies (excludes Cubans) in Broward County. Haitians made up the largest population of immigrants (48%), with Jamaicans at 41% [7]. The leading causes of death for this population include heart disease, cancer, stroke, and diabetes – all impacted by diet and exercise [3]. Proper nutrition offers one of the most effective ways to decrease the risk factors associated with the burden of many poor health outcomes, chronic conditions, and diseases. Since obesity and related complications (diabetes, heart disease, hypertension) continue to rise in South Florida, it is imperative to reach every demographic of our ethnically diverse community with culturally appropriate educational materials to achieve Healthy People 2020 and 2030 national goals.

Given the large number of Caribbean-born residents, Caribbean heritage families in South Florida, and those not accounted for in the census data, there is a strong need to provide culturally appropriate nutrition and healthy food options. To our knowledge, our NSU Quality of Life Grant Funded Project, the Caribbean Diaspora Healthy Nutrition Outreach Project (CDHNOP), is the first, research-driven, educational outreach campaign tailored to be culturally appropriate for the Caribbean diaspora using the format of the *Go Slow Whoa*, or GSW [9]. The goal of this Initiative is to create, disseminate and test the effectiveness of educational materials tailored to Caribbean diaspora. Using a focus group research frame, informational and educational materials were developed using evidence-based strategies to provide a straightforward categorization system that helps people make healthier choices. The Coordinated Approach to Child Health’s (CATCH) *Go–Slow–Whoa* is a tool to guide children and families toward making healthful food choices [8]. The overall message is that all foods can fit into a healthy diet, which consists of more *Go* foods than *Slow* foods, and more *Slow* foods than WHOA foods. The evidence-based “Go Slow Whoa” (GSW) intervention provides the public with guidelines that designate whether a food or beverage should be chosen frequently (Go, colored green), less often (Slow, colored yellow), or rarely (Whoa, colored red) based on the content of nutrients, sodium, fat and sugar [8]. This system is designed to be used in a variety of food service areas, including retail outlets, restaurants, vending machines and cafeterias. Materials are brightly colored and include images of food to help people find healthy items. These decals, posters, graphics, and handouts describe the GSW categories and inform patrons of the benefits of choice.

## Objectives and Statement of Need

There are 2 main objectives: 1) creating and evaluating culturally appropriate nutrition materials for the Caribbean diaspora population in Broward County (i.e. tailor existing evidence–based materials for this population), and 2) to quantify which social determinants of health are most relevant to this population. We captured information on social determinants of health using questions extracted from the PRAPARE Assessment tool. The Protocol for Responding to and Assessing Patients’ Assets, Risks, and Experiences (PRAPARE) is a national effort to help health centers and other clinicians collect the data needed to better understand and act on their patients’ social determinants of health [9]. This aligns with our population health goals while reducing costs. This will also allow us to identify the upstream socioeconomic drivers of poor outcomes and higher costs. With data on the social determinants of health, we can define and document the increased complexity of our patients, transform care with integrated services, and build strategic community partnerships that bring value to our heterogeneous community.

## Overall Methods

This study employed mixed methods inclusive of a qualitative focus group research frame and a quantitative collection of social determinants of health and intercept survey data.

## Focus Group Methods

In order to gain a contextual understanding of mis- and under-representation of Caribbean culture in present nutrition materials, an in-depth analysis was performed via semi-structured focus groups during which both quantitative and qualitative data were collected from participants from the top five Caribbean islands represented in our geographic location: Haiti, Jamaica, Dominican Republic, Cuba and Trinidad and Tobago. The focus group provides a research frame that includes data collection, data reduction, and data grouping, inference, analysis, validation, and results which test the hypothesis that these modified GSW materials will encourage healthy eating. The data received through this process and the content analysis provide the information required to: answer the research question, determine which items to modify, and provide the basis for the proposed educational materials and activities. The data analysis process included data grouping, where the answers from all of the focus groups are combined; information labelling that shows what each group of answers describe, which items resonate with participants and where there is dissonance; knowledge and the findings, and, the implications of what is learned from modifications, preferences and language. The use of the focus group research frame allowed the team to describe, gain a better understanding, and determine directions in the development, translation, and modification of new materials. Participants (Cuba = 8, Dominican Republic = 7, Haiti = 8, Jamaica = 7, and Trinidad and Tobago = 8) attended one of seven focus groups. Although five focus groups stratified by island of origin were planned, two additional groups were added in order to accommodate participants who were unable to attend the focus group they were initially selected for. Subjects were eligible if they identified as a member of the Caribbean diaspora from one of the five Caribbean countries described above, 18 years of age or older, if they could read and speak English and if they lived in Broward County for at least five years. All our focus groups and surveys were conducted using the English language due to the availability of translated materials and study staff qualifications. Our study team chose five years living in Broward County as an inclusion criteria to allow our participants who lived this long in Broward County to have a better understanding and use of Broward County resources since they answered questions related to this in our social determinants survey. Future planned studies will remove the restriction on the number of years living in Broward County to include data on recent immigrants. In addition, to be more inclusive to Caribbean immigrants, our future studies will include a mix of Caribbean language options such as Haitian Creole, Spanish and English. We targeted three zip codes that have a high concentration of Caribbean residents: 33311, 33313, and 33319. Participants were recruited using purposive sampling as well as snowball sampling. This allowed researchers to identify fitting candidates for each focus group (purposive sampling), but also ensured variability and representability (snowball sampling). Purposive sampling was done by the Principal Investigator to ensure that all 5 Caribbean islands were well represented among participants and that participants were not from the same family. Some participants came from mixed Caribbean island backgrounds. They were asked which island they most identified with before being assessed for further study eligibility. This was done in the eligibility sessions that occurred before each of the focus groups to ensure that focus groups could be accurately stratified by Caribbean island of origin. In order to maintain anonymity and de-identification of participants as aligned with our IRB requirements and exempt status, no audio recordings were performed. In addition, to gain more trust with our Caribbean community, we decided against recording our sessions to encourage engagement and unrestricted participation of our focus group participants. Notetakers included the PI and an assigned study member who either handwrote or typed notes to be used later for coding and analysis. Additionally, notes were taken on large Flip boards visible to all participants to further ensure an accurate representation of their ideas. The structure of the focus group discussion was guided by pre-written questions as well as the five packets of existing *Go-Slow-Whoa* health and nutrition information flyers. Participants were asked to evaluate each of the packets. The discussion for each packet began with participants providing the page of the packet that they identified most with. Guided by the moderator, the group then discussed every page of every packet to identify missing foods, cultural notions, and organization of information. Throughout each focus group, participants mentioned food items, meals and exercise activities in present nutrition and active living material that were either absent or did not have cultural meaning. Additionally, participants provided insight as to food items and meals that are common in their respective cultures to be included in future material tailored to Caribbean culture. At the end of each focus group, participants were asked to provide names of community centers, restaurants, grocery stores and other places that they frequented. This information allowed us to identify key sites in which to distribute program materials and that can serve as locations for educational forums to demonstrate healthy cooking demonstrations and nutrition/active living education.

Focus group notes were hand-coded to identify overarching themes and to organize cultural insights provided from participants in each group. Our grant partners for this project were many of the same community stakeholders who participated in the original Broward County Go Slow Whoa educational campaign that was geared towards the general community. Nutrition experts from among our team of grant partners reviewed the food, nutrition details and ensured that our final Caribbean-centered education materials were accurate and evidence-based. Final education materials were presented to the Broward Regional Health Planning Council’s Health Care Access Committee and the Broward County Department of Health’s Nutrition and Fitness Task Force for final review. These final materials reflected the combined preferences of all five Caribbean countries and were translated in English, Spanish and Creole.

## Focus Group Quantitative Survey Methods

As this was an exploratory study, we employed both descriptive and inferential statistics. Demographic and quantitative data were collected with the use of a 20-item survey adapted from the PRAPARE survey set. The survey’s content validity was assessed by experts in the areas of survey and health disparities research. Descriptive statistics were calculated for all study variables. This included counts and percentages for categorical variables, mean and standard deviations for continuous measures. Fisher’s exact test was used to examine associations between the country of origin (Cuba, Dominican Republic, Haiti, Jamaica, and Trinidad and Tobago) and outcome variables such as education, employment, stress level, employment, and someone to speak with about health care. The statistical package R 3.4.4 was used in all analyses, and statistical significance was found at p < 0.05.

## Intercept Survey Methods

We performed preliminary intercept surveys to gauge the effectiveness of our materials. A convenience sample of 24 Caribbean participants in Broward County who did not participate in our focus groups completed our intercept surveys: Haiti (3); Jamaica (7); Dominican Republic (4); Trinidad and Tobago (7); Cuba (3) (see Table 4). These intercept surveys were done at various locations that displayed our educational materials or performed individually after each participant had time to review a booklet of our education materials immediately prior to taking this survey.

## Results

### Qualitative Focus Group Results

Participants attended focus groups based on the Caribbean island with which they identified most with. Stratification by island was done intentionally to foster uninhibited participation and reduce bias. During analysis, however, recurring themes became apparent – participants from different focus groups expressed similar ideas, especially when it came to food items and cultural insight. Furthermore, the intent of this project is to holistically assess the Caribbean diaspora population in South Florida; this was only echoed by what researchers found as immense Caribbean cultural integration. Thus, it was most appropriate to combine results from all Caribbean countries in this study. Results are displayed in Tables 1 and 2.

**Table 1:** Food and Exercise Themes Identified by Focus Groups in Broward County, FL.

Category	Food Item

Fruits	Mango Guava Avocado Papaya Soursop Pineapple
Meat and Seafood	Chicken (stew, curry) Goat Oxtail Pork Salami Snapper Cod Eggs Shrimp
Vegetables and Beans	Cabbage Plantains Corn Pumpkin (butternut squash) Kidney beans Beans Pigeon peas
Wheat and Grains	Bread Roti Cream of wheat Baked mac & cheese (pasta) Rice (Jasmine, Basmati)
Drinks	Fresh fruit juice (natural, with added milk or condensed milk and brown sugar) Coffee (sometimes black, sometimes with cream and sugar) Colas (respective to island and/or American brands such as Pepsi and Coca-Cola products) Malta
Exercise	American football and swimming in pools are unrelatable and/or unpopular for exercise Dancing, baseball, cricket, and soccer are common for exercise and for entertainment Walking (in groups, with families, outdoors) is favored by all groups for exercise

**Table 2:** Cultural Themes Gathered from Focus Group Participants in Broward County, FL 2018.

Theme	Details

Organization	Colors: participants value bright colors and color-coded information Layout: bulleted information is easier to understand and more attractive; cluttered information is useless and can be easily ignored; logos repeatedly reduce visual fluidity Phrases: key terms should be bolded, and catchy slogans/phrases are favorable to participants
Information	Exclude overused material (apple equals health, food pyramid, etc.) Calorie counts and nutritional information are highly valued Food equations (ex. 1 donut hole = 27 grapes) should be comparable (snacks compared to snacks, meals compared to meals, etc.) Include culturally appropriate exercises Swimming among Afro-Caribbean American women is uncommon Walking is favored by all groups and is extremely popular
Illustrations, Pictures, and Graphics	Participants from all groups favor depictions of families (exercising) and happy children Crowded graphics are disliked (ex: My Plate Planner) Graphics should be clear and easy to understand (Page 2 of My Plate Planner was often mistaken for sign language) Doughnut holes (Colors of Health packet), candy bar (Soda? Think Again packet), and soda cans (Soda? Think Again packet) are not easily recognizable to many participants Inclusion of cultural items (dominos, wooden spoons) is encouraging and more representative Some items (i.e. hockey pucks) are uncommon in Caribbean culture
Cultural Insights	Eggs are common in Caribbean cuisine, but the nutritional value of eggs is often misinterpreted. Many believe that eggs are bad for you, so nutritional information about eggs is useful Almonds aren’t popular – peanuts are more popular Portion size/control isn’t popular or widely understood Canned food isn’t considered healthy Consider using more culturally appropriate foods for comparison and inclusion Colors of Health Packet – blueberries can be equated to sickness/medicine and negatively received Sports and exercise: baseball, cricket, dancing, and soccer are more popular than American football Some cultures commonly indulge in spicy foods (especially use of scotch bonnet peppers) and others are impartial

### Focus Group Quantitative Survey Results

The average age of all participants was 43.1 (SD = 13.4), the median number of family members was 3 (IQR = 1–6), 39% self-identified as Black or African American, 79% were female, 87% possessed more than a high school diploma, and 82% were employed full-time.

Bivariate analysis revealed employment was significantly associated with country of origin. More individuals from Haiti (38%) and Trinidad and Tobago (50%) were unemployed than individuals from Cuba, Dominican Republic or Jamaica (p < 0.05). No other variable was significantly associated with country of origin — Table 3.

**Table 3:** Participant Demographics and Social Determinants of Health Reported by Focus Group Participants in Broward County, FL.

Country	Gender			P-Value

	Female		Male	

Cuba	6 (75.0)		2 (25.0)	p = 0.714
Dominican Republic	6 (85.7)		1 (14.3)	
Haiti	7 (87.5)		1 (12.5)	
Jamaica	6 (85.7)		1 (14.3)	
Trinidad and Tobago	5 (62.5)		3 (37.5)	
**Country**	**Race**			**P-Value**

	**Black/AA**		**Other**	

Cuba	1 (14.3)		6 (85.7)	p = 0.019
Dominican Republic	2 (28.6)		5 (71.4)	
Haiti	6 (75.0)		2 (25.0)	
Jamaica	5 (71.4)		2 (28.6)	
Trinidad and Tobago	1 (12.5)		7 (87.5)	
**Country**	**Education**			**P-Value**

	**High School/GED**		**More than High School**	

Cuba	1 (12.5)		7 (87.5)	p = 0.400
Dominican Republic	0 (0.0)		7 (100.0)	
Haiti	0 (0.0)		8 (100.0)	
Jamaica	1 (16.7)		5 (83.3)	
Trinidad and Tobago	2 (25.0)		6 (75.0)	
**Country**	**Employment**			**P-Value**

	**Employed**		**Unemployed**	

Cuba	8 (100.0)		0 (0.0)	p = 0.015
Dominican Republic	7 (100.0)		0 (0.0)	
Haiti	5 (62.5)		3 (37.5)	
Jamaica	7 (100.0)		0 (0.0)	
Trinidad and Tobago	4 (50.0)		4 (50.0)	
**Country**	**Stressed**^1^			**P-Value**

	**No**		**Yes**	

Cuba	6 (75.0)		2 (25.0)	p = 0.594
Dominican Republic	3 (42.9)		4 (57.1)	
Haiti	6 (75.0)		2 (25.0)	
Jamaica	4 (66.7)		2 (33.3)	
Trinidad and Tobago	4 (50.0)		4 (50.0)	
**Country**	**Jobs**			**P-Value**

	**Zero**	**One**	**Two**	

Cuba	0 (0.0)	6 (75.0)	2 (25.0)	p = 0.134
Dominican Republic	0 (0.0)	6 (85.7)	1 (14.3)	
Haiti	3 (37.5)	4 (50.0)	1 (12.5)	
Jamaica	0 (0.0)	5 (71.4)	2 (28.6)	
Trinidad and Tobago	4 (50.0)	3 (37.5)	1 (12.5)	
**Country**	**Summary Statistics (Continued) Talking^2^**			**P-Value**

	**2 or less per week**	**3–5 Times Week**	**5+ Times a Week**	

Cuba	2 (25.0)	3 (37.5)	3 (37.5)	p = 0.704
Dominican Republic	1 (14.3)	3 (42.9)	3 (42.9)	
Haiti	3 (37.5)	3 (37.5)	2 (25.0)	
Jamaica	2 (33.3)	0 (0.0)	4 (66.7)	
Trinidad and Tobago	1 (12.5)	3 (37.5)	4 (50.0)	

^1^ “Stressed” here refers to the PRAPARE survey item that asked participants the following: Are you stressed (Yes vs No)? ^2^ “Talking” here refers to the PRAPARE survey item that asked participants the following: How often do you talk to people that you care about and feel close to (2 or less per week, 3–5 times per week, 5+ times per week)?

#### Access to Healthcare

**Figure 1:**
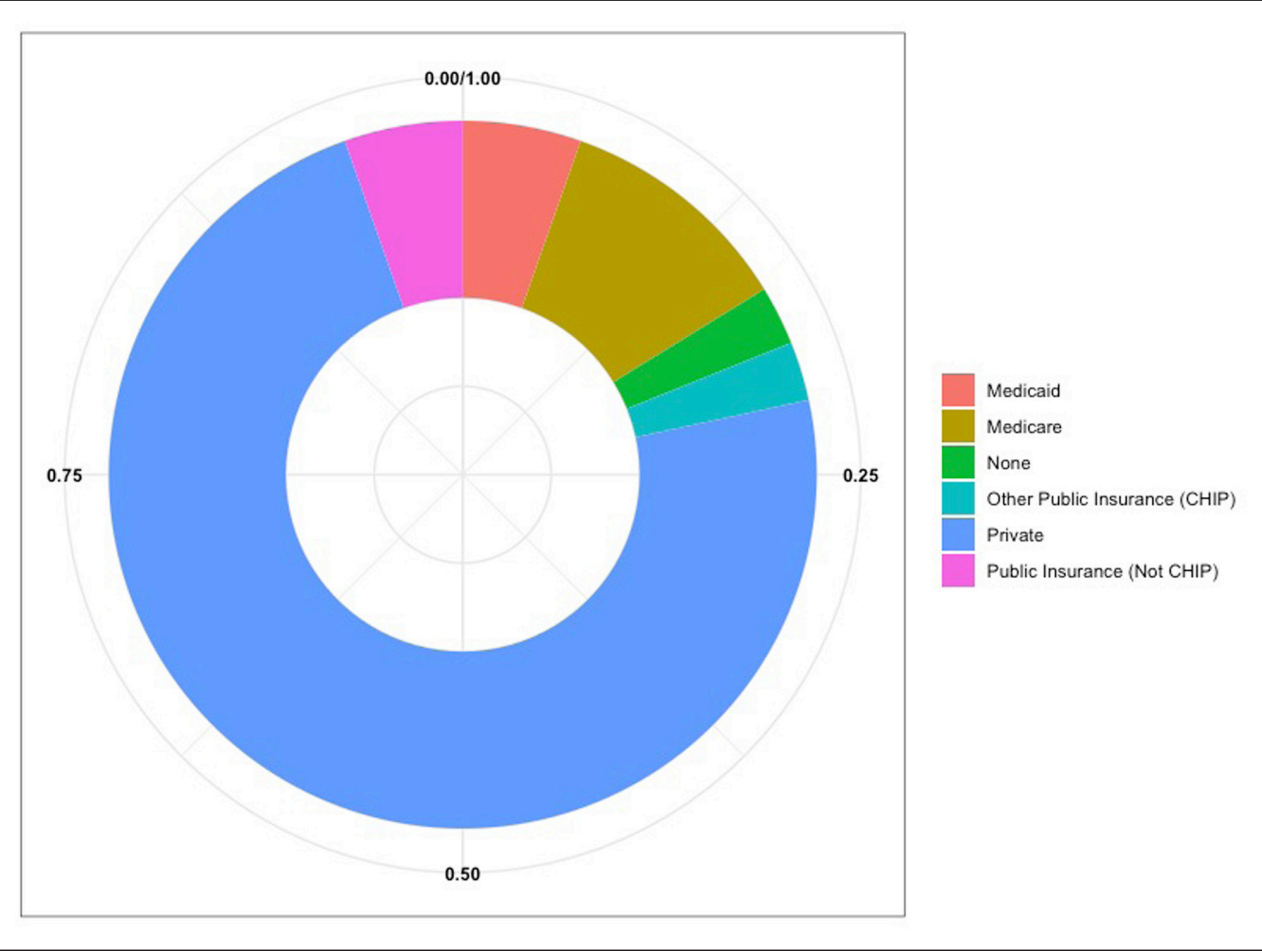
Insurance.

**Figure 2:**
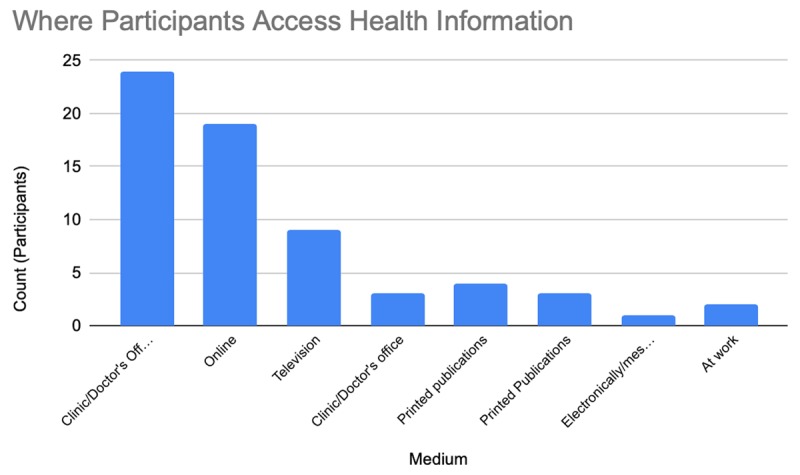
How Participants Access Health Information.

**Figure 3:**
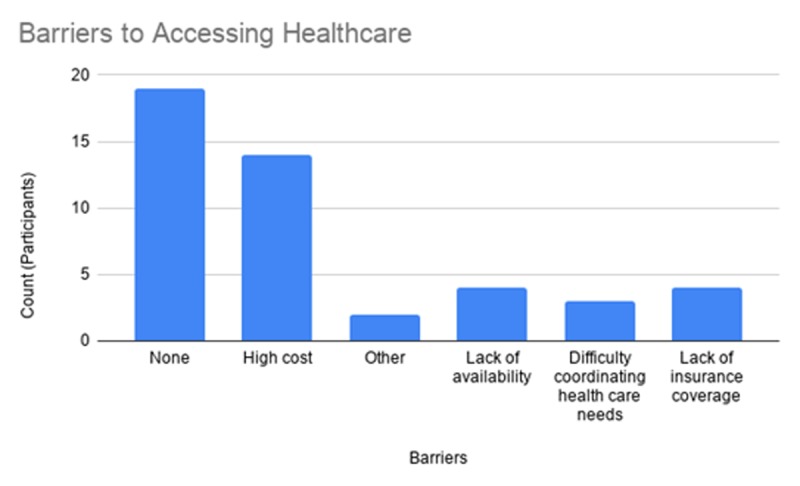
Barriers to Accessing Health Care.

Access was measured using responses to the following four questions:

1. What is your main insurance (Public vs Private)? (Figure 1)
2. Has lack of transportation kept you from medical appointments, meetings, work, or from getting things needed for daily living (Yes vs No)?
3. How do you receive health information (clinic/doctor’s office, printed publications, electroni-cally/message, community resources, online, at work, NSU Health Office)? (Figure 2)
4. Do you face barriers accessing health services (Participants selected one option from a list)? (Figure 3)

Seventy-three percent of respondents obtain insurance through their employer (private), 92% have adequate transportation for daily living, 66% receive health information from their doctor. Although 50% of participants reported no barriers in access to care, the primary barrier was cost among participants who faced a barrier (47%).

Figure 2: Most participants (65.8%) report that they primarily access health information at a clinic or doctor’s office. A total of 26.3% of participants access health information online (via Google, WebMD, Mayo Clinic, or NSU Health Benefits online, or another website).

Figure 3: While half of the participants (50%) face no barriers accessing healthcare, half of participants face at least one barrier. The most common barrier is the high cost of health care (expressed by 34.2% of participants).

#### Housing

Housing was measured using responses to the following three questions:

1. How many family members do you currently live with?
2. What is your housing situation today (Unsheltered vs Sheltered)?
3. Do you have trouble paying for housing or your electric/heating bills (Yes vs No)?

The median number of family members was 3 (range 1–6), 92% of respondents possessed housing, and 92% do not have trouble paying for housing or electrical/heating bills.

#### Stress and Safety

Stress and safety were measured using responses to the following three questions:

1. Do you feel physically and emotionally safe where you currently live (Yes vs No)?
2. How often do you talk to people that you care about and feel close to (2 or less per week, 3–5 times per week, 5+ times per week)?
3. Are you stressed (Yes vs No)?

Eighty-four percent of respondents feel safe in their community, 42% speak with someone they care about five or more times a day, and 37% are stressed.

#### Food Access

Food access was measured using response to two questions:

1. Do you ever have time during the month when you do not have enough to eat (Yes vs No)?
2. In the past year, have you or any family members been unable to get food (Yes vs No)?

Eighty-two percent of respondents always had enough to eat and, 92% were able to get food.

### Intercept Survey Results

The majority of participants noted taste, as opposed to fat and sugar content as important factors in selecting food/beverages in a restaurant or store. At 92% and 82% respectively, an overwhelming majority of our surveys indicated that our newly created healthy eating, active living materials were helpful in making positive food and beverage choices. In addition, 83% and 84% of respondents respectively noted that our education materials influenced their food and beverage choices. As this was a small sample, future intercept surveys will include a larger number of participants (Table 4).

**Table 4:** Intercept Survey Data.

Question 1 Response	On average, how many times do you go to the restaurant/store for food and beverages per week?					

	Haiti	Jamaica	Dominican Republic	Trinidad & Tobago	Cuba	Total

Less than once per week	1	1	2	2		25%
1–2 times/week	2	2	1	3	3	46%
3–5 times/week		4		2		25%
>5 times/week			1			4%
**Question 2 Response**	What is most important to you when making your food or beverage selections in the restaurant/store?[*]					

	**Haiti**	**Jamaica**	**Dominican Republic**	**Trinidad & Tobago**	**Cuba**	

Taste	2	5	1	5		
Cost			1	2	2	
Low Fat Content			1			
Low Sugar Content			1	1		
High Nutritional Content	1	2	2	1	2	
**Question 3 Response**	**Did you find the Go, Slow, Whoa posters or brochures helpful in making beverage choices?**					

	**Haiti**	**Jamaica**	**Dominican Republic**	**Trinidad & Tobago**	**Cuba**	**Total**

Yes	3	7	3	6	3	92%
No			1	1		8%
Maybe						0%
**Question 4 Response**	**Did the Go, Slow, Whoa posters or brochures influence your beverage choices?**					

	**Haiti**	**Jamaica**	**Dominican Republic**	**Trinidad & Tobago**	**Cuba**	**Total**

Yes	3	7	2	5	3	84%
No			1	1		8%
Maybe			1	1		8%
**Question 5 Response**	**Did you find the Go, Slow, Whoa posters or brochures helpful in making food choices?**					

	**Haiti**	**Jamaica**	**Dominican Republic**	**Trinidad & Tobago**	**Cuba**	**Total**

Yes	3	7	4	5	3	92%
No				2		8%
Maybe						0%
**Question 6 Response**	**Did the Go, Slow, Whoa posters or brochures influence your food choices?**					

	**Haiti**	**Jamaica**	**Dominican Republic**	**Trinidad & Tobago**	**Cuba**	**Total**

Yes	3	7	3	4	3	83%
No			1	3		17%
Maybe						0%

[*] Multiple Response Question.

## Discussion

Obesity prevention and its associated co-morbidities such as diabetes, heart disease and stroke require a multi-tiered, culturally sensitive, population-based approach. An inclusive, relationship building approach to healthy nutrition, active living education and research is more likely to be embraced from the diverse communities we serve. Given the dearth of disaggregated data on Caribbean immigrants, the significance of this project reinforces the success of a community-based participatory research model and adds another dimension of much needed culturally appropriate healthy living education for the Caribbean diaspora. A critical component of the CDHNOP project is that it has built trust between the local Caribbean community and key personnel of this project. This puts the project team in a unique position to explore health disparities further in this population and to gain momentum in developing more reliable, comprehensive, actionable data. Our preliminary intercept survey results suggest a great need for these interventions and reflects the importance of how cultural factors impact the health and wellness of our community. Our social determinants of health data suggest that most Caribbean immigrants receive health information from their doctor. The average age of our participants, 43.1, aligns with Generation X who typically appreciate convenient access to healthcare [10]. Our study suggests that a Caribbean patient’s clinician is a trusted source of health information despite the challenges of convenience and cost. In addition, since most of our participants were female (79%), further inquiry into the role of women in Caribbean households with regards to income, healthcare decisions and other related social determinants of health require further exploration. Of note, 37% of our study subjects considered themselves stressed with 42% able to speak to someone they care about 5 or more times a day. Exploring cultural factors that influence mental health assistance in our Caribbean community will shed light on other variables that impact overall health and wellness in this understudied population. A national study found that the small number of Blacks of Caribbean ancestry included in the sample had higher levels of psychological stress compared with US-born Blacks [11,12]. These factors and variables require further exploration in order to make a significant positive impact on the health and lives of our ever-growing Caribbean community.

## Limitations

This study is not without its limitations. Unintentionally inflated levels of impartiality and bias as compared to quantitative studies is a common disadvantage among studies that employ qualitative methods [13]. In addition, variability in the study population may lead to findings that are non-representative of the population [14]. This study included individuals who, for the most part, were educated, insured with private insurance, and experienced little to no stress (demographics gathered by the PRAPARE survey participants completed). This study is also limited by the small sample size. In future studies, the research team plans to incorporate a larger, more robust sample in order to include a more diverse, representative sample. Another limitation was the use of handwritten or typed notes instead of audio recordings with transcription. While our study team built significant trust with the Caribbean community from this method, we are subject to human error in capturing every spoken detail from these focus groups. Lastly, our study team spoke primarily English and materials were only available in the English language. Future studies will include study team members proficient in languages such as English, Spanish and Haitian Creole. Study materials will also be available in English, Spanish and Haitian Creole.

## Conclusions

Public health research is the basis on which intervention is planned and executed, policy changes rely on, and epidemiologic patterns are established. Our preliminary literature review demonstrated the lack of purposeful inclusion of the Caribbean diaspora. CDHNOP aims to address these shortcomings by identifying important patterns of dietary and exercise preferences within this population. Several of the most prevalent diseases and conditions within the Caribbean population (diabetes, cardiovascular disease, obesity) are associated with nutritional patterns, deeming these previously unidentified patterns essential to understanding the most plaguing morbidities within the geographic group. This unparalleled research aims to contribute to one of several crucial pillars necessary to understanding health-related patterns within the Caribbean diaspora. One of the most important qualitative findings of this study is that the Caribbean sub-population of South Florida largely feels underrepresented in *GSW* material. Making the language and graphics more inclusive of Caribbean dietary and exercise preferences, will help promote positive healthy behaviors in innovative ways.

To access materials created from this project and for more information, visit https://md.nova.edu/community-health/caribbean-health.html.

## Additional Files

The additional files for this article can be found as follows:

10.5334/aogh.2657.s1 Caribbean Diaspora Healthy Nutrition Outreach Project Quantitative Analysis. A brief exploration (including graphics and summarizing tables) of quantitative statistical analysis of data gathered as part of CDHNOP data collection.

10.5334/aogh.2657.s2 Caribbean Diaspora Healthy Nutrition Outreach Project Results. Graphic representation of select quantitative and qualitative data points included in the CDHNOP dataset.
