# Supplementary material for: Caribbean Diaspora Healthy Nutrition Outreach Project (CDHNOP): A Qualitative and Quantitative Approach to Caribbean Health
Source: Ann Glob Health. 2020 Feb 4;86(1):12. doi: 10.5334/aogh.2657 (PMC7006587; doi:10.5334/aogh.2657)
Supplement: Caribbean Diaspora Healthy Nutrition Outreach Project Quantitative Analysis. — A brief exploration (including graphics and summarizing tables) of quantitative statistical analysis of data gathered as part of CDHNOP data collection. [file agh-86-1-2657-s1.pdf]

Methodology

As this was an exploratory study we employed both descriptive and inferential statistics. Descriptive statistics were calculated for all study variable. This included counts and percentages for categorical variables, mean and standard deviations for continuous measures. To examine associations between country of origin (Cuba, Dominican Republic, Haiti, Jamaica, and Trinidad and Tobago) and the outcome variables education, employment, stress level, employment, and someone to speak with about health care, Fisher's exact test was used. The statistical package R 3.4.4 was used in all analyses and statistical significance was found at $p < 0.05$.

Results

Participants were from five Caribbean countries (Cuba = 8, Dominican Republic = 7, Haiti = 8, Jamaica = 7, and Trinidad and Tobago = 8), The average age of all participants was 43.1 (SD =13.4), the median number of family members was 3 (IQR = 1 - 6), 39% self-identified as Black or African American, 79% were female, 87% possessed more than a high school diploma and 82% were employed full-time.

Bivariate analysis revealed employment was significantly associated with country of origin. More individuals from Haiti (38%) and Trinidad and Tobago (50%) were unemployed than individuals from Cuba, Dominican Republic or Jamaica ($p < 0.05$). No other variable was significantly associated with country of origin —Table 1.

Access

Access was measured using responses to the following four questions:

1. What is your main insurance (Public vs Private)?
2. Has lack of transportation kept you from medical appointments (Yes vs No)?
3. How do you receive health information (Doctor vs Other)?
4. Do you face barriers accessing health services (Yes vs No)?

Seventy-three percent of respondents obtain insurance through their employer (private), 92% have adequate transportation for daily living, 66% receive health information from their doctor, and the primary barrier to access was cost (47%). Figure 1 provides an alluvial plot for the relationship between island, barrier and insurance type.

Housing

Housing was measured using responses to the following three questions:

1. How many family members do you currently live with?
2. What is your housing situation today (Unsheltered vs Sheltered)?
3. Do you have trouble paying for housing or your electric/heating bills (Yes vs No)?

The median number of family members was 3 (range 1 - 6), 92% of respondents possessed. housing, and 92% do not have trouble paying for housing or electrical/heating bills. Figure 2 provides an alluvial plot for the relationship between the number of people in a household, housing situation, and trouble paying for housing or electrical/heating bills.

Stress and Safety

Stress and safety was measured using responses to the following three questions:

1. Do you feel physically and emotionally safe where you currently live (Yes vs No)?
2. How often do you talk to people that you care about and feel close to (2 or less per week, 3-5 times per week, 5+ times per week)?
3. Are you stressed (Yes, vs No)?

Eighty-four percent of respondents feel safe in their community, 42% speak with someone they care about five or more times a day, and 37% are stressed. Figure 3 provides an alluvial plot for the relationship between safety, number of people to speak with, and stress—Figure 3.

Food Access

Food access was measured using response to two questions:

1. Do you ever have time during the month when you do not have enough to eat (Yes vs No)?
2. In the past year, have you or any family members been unable to get food (Yes, vs No)?

Eighty-two percent of respondents always had enough to eat and , 92% were able to get food. Figure 4 provides an alluvial plot for the relationship between island, enough to eat and unable to get food—Figure 4.

Table 1: Summary Statistics

	Gender		
**Country**	**Female**	**Male**	**P-Value**
Cuba	6 (75.0)	2 (25.0)	p = 0.714
Dominican Republic	6 (85.7)	1 (14.3)	
Haiti	7 (87.5)	1 (12.5)	
Jamaica	6 (85.7)	1 (14.3)	
Trinidad and Tobago	5 (62.5)	3 (37.5)	
	**Race**		
**Country**	**Black/AA**	**Other**	**P-Value**
Cuba	1 (14.3)	6 (85.7)	p = 0.019
Dominican Republic	2 (28.6)	5 (71.4)	
Haiti	6 (75.0)	2 (25.0)	
Jamaica	5 (71.4)	2 (28.6)	
Trinidad and Tobago	1 (12.5)	7 (87.5)	
	**Education**		
**Country**	**High School/GED**	**More than High School**	**P-Value**
Cuba	1 (12.5)	7 (87.5)	p = 0.400
Dominican Republic	0 (0.0)	7 (100.0)	
Haiti	0 (0.0)	8 (100.0)	
Jamaica	1 (16.7)	5 (83.3)	
Trinidad and Tobago	2 (25.0)	6 (75.0)	
	**Employment**		
**Country**	**Employed**	**Unemployed**	**P-Value**
Cuba	8 (100.0)	0 (0.0)	p = 0.015
Dominican Republic	7 (100.0)	0 (0.0)	
Haiti	5 (62.5)	3 (37.5)	
Jamaica	7 (100.0)	0 (0.0)	
Trinidad and Tobago	4 (50.0)	4 (50.0)	

Table 2: Summary statistics (continued)

	Stressed		
**Country**	**No**	**Yes**	**P-Value**
Cuba	6 (75.0)	2 (25.0)	p = 0.594
Dominican Republic	3 (42.9)	4 (57.1)	
Haiti	6 (75.0)	2 (25.0)	
Jamaica	4 (66.7)	2 (33.3)	
Trinidad and Tobago	4 (50.0)	4 (50.0)	

	Jobs			
**Country**	**Zero**	**One**	**Two**	**P-Value**
Cuba	0 (0.0)	6 (75.0)	2 (25.0)	p = 0.134
Dominican Republic	0 (0.0)	6 (85.7)	1 (14.3)	
Haiti	3 (37.5)	4 (50.0)	1 (12.5)	
Jamaica	0 (0.0)	5 (71.4)	2 (28.6)	
Trinidad and Tobago	4 (50.0)	3 (37.5)	1 (12.5)	

	Talking			
**Country**	**2 or less per week**	**3 - 5 Times Week**	**5+ Times a Week**	**P-Value**
Cuba	2 (25.0)	3 (37.5)	3 (37.5)	p = 0.704
Dominican Republic	1 (14.3)	3 (42.9)	3 (42.9)	
Haiti	3 (37.5)	3 (37.5)	2 (25.0)	
Jamaica	2 (33.3)	0 (0.0)	4 (66.7)	
Trinidad and Tobago	1 (12.5)	3 (37.5)	4 (50.0)	

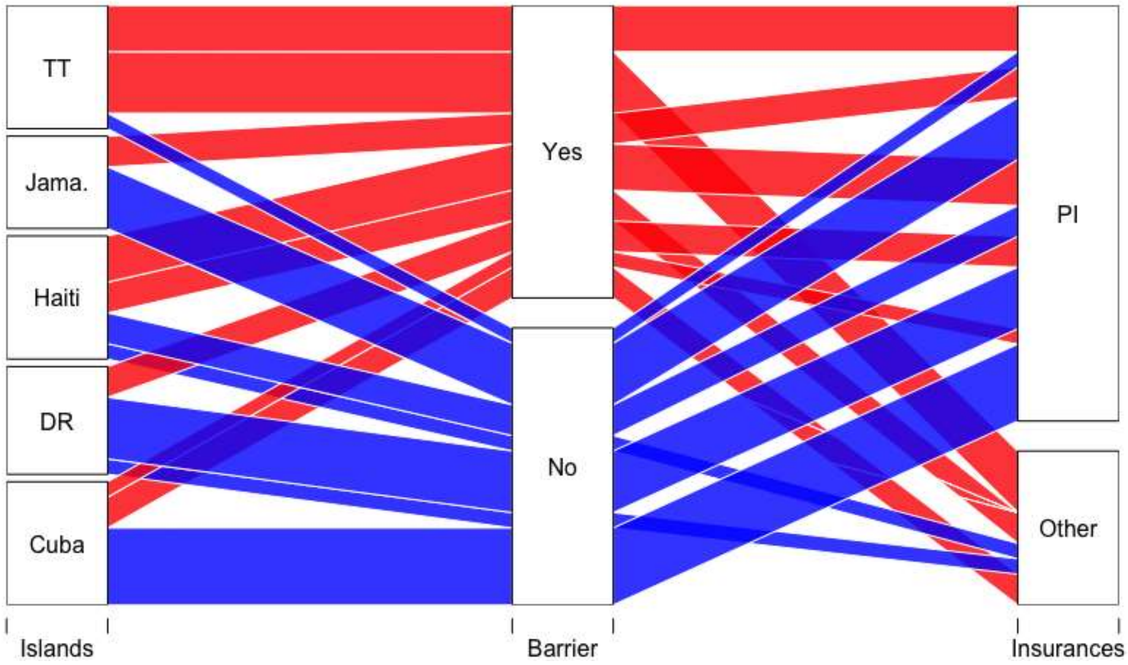

Figure 1. Alluvial plot of Island, barriers and insurance type.

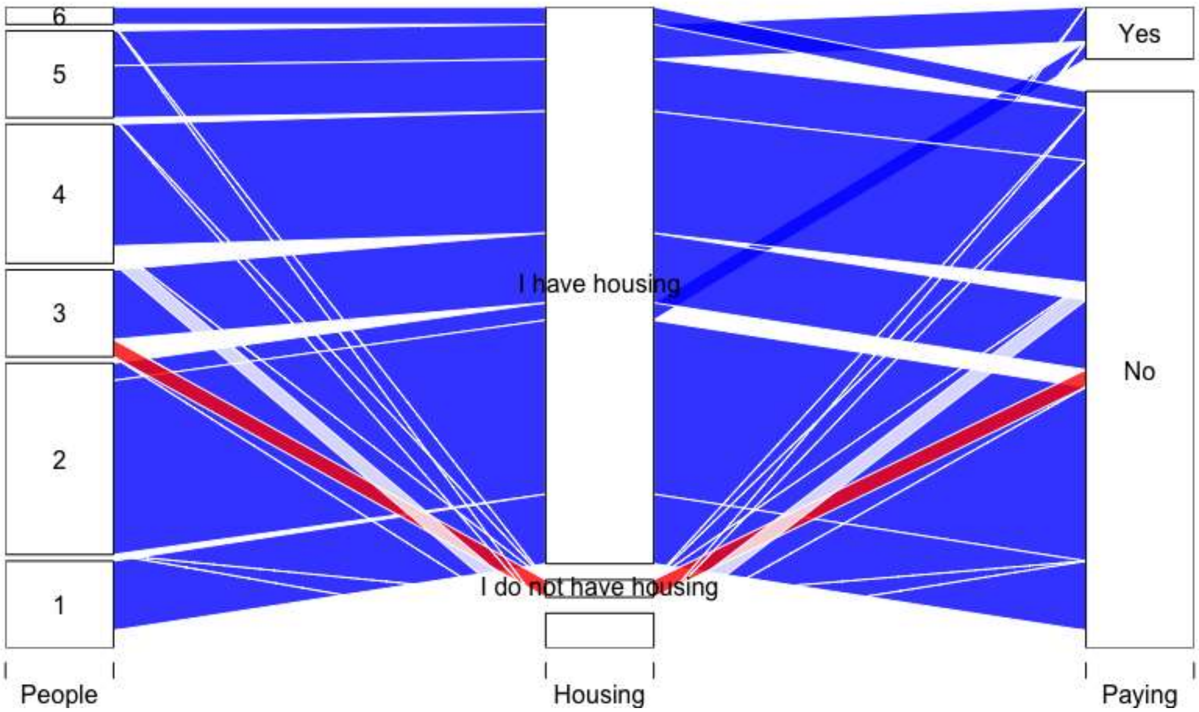

Figure 2. Alluvial plot for the number in household, housing and paying for housing.

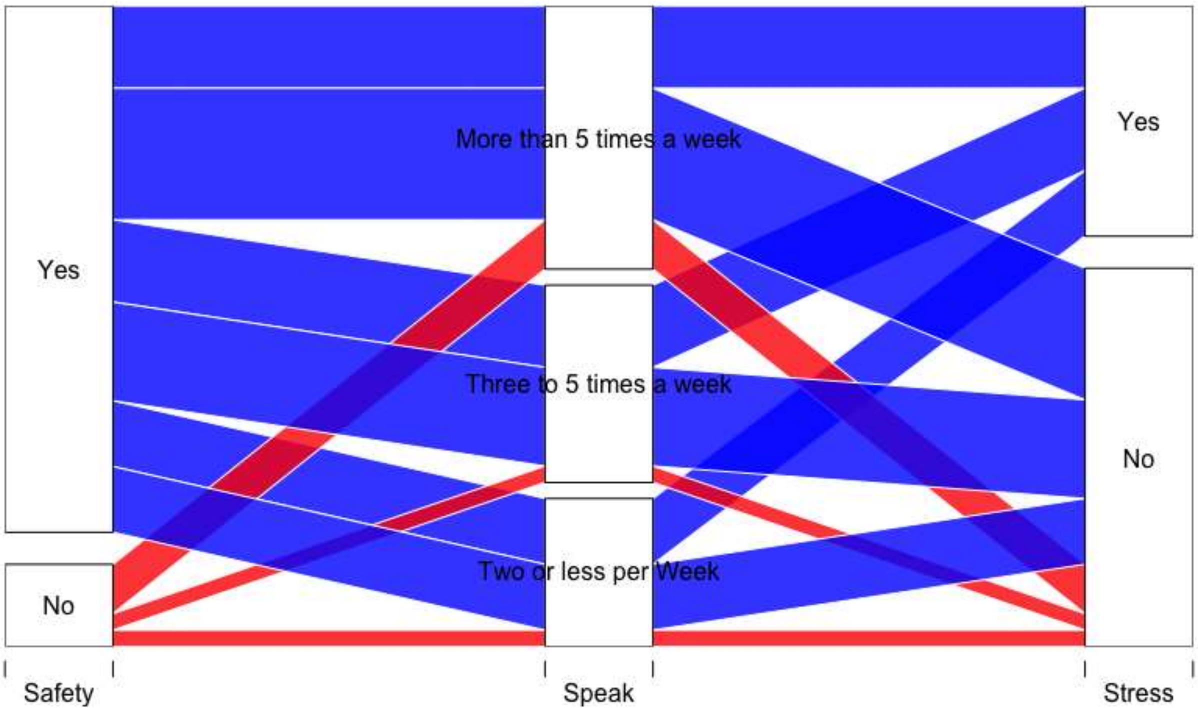

Figure 3. Alluvial plot for safety, speak, and stress.

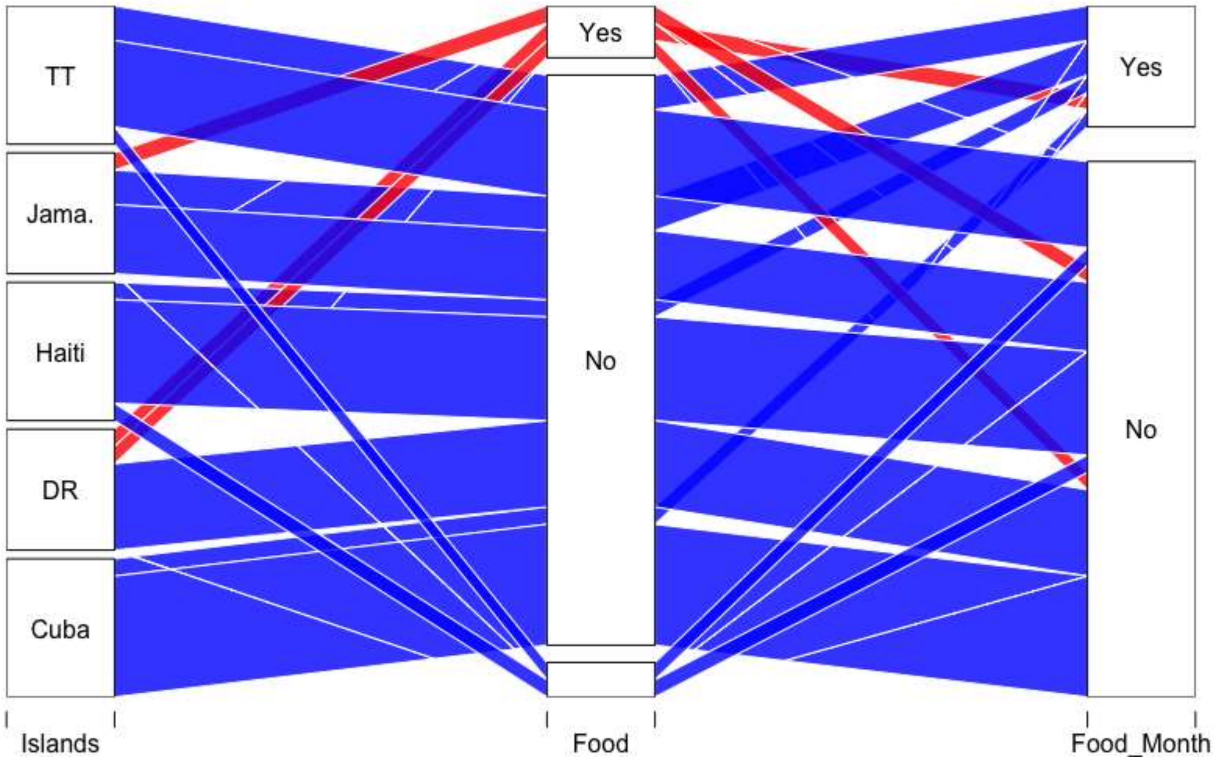

Figure 4. Alluvial plot for island, not have enough to eat, and unable to get food
