# Supplementary material for: Caribbean Diaspora Healthy Nutrition Outreach Project (CDHNOP): A Qualitative and Quantitative Approach to Caribbean Health
Source: Ann Glob Health. 2020 Feb 4;86(1):12. doi: 10.5334/aogh.2657 (PMC7006587; doi:10.5334/aogh.2657)
Supplement: Caribbean Diaspora Healthy Nutrition Outreach Project Results. — Graphic representation of select quantitative and qualitative data points included in the CDHNOP dataset. [file agh-86-1-2657-s2.pdf]

Caribbean Diaspora Healthy Nutrition Outreach Project Results

Data Analysis

Surveys entitled "Social Determinants of Health" were used to collect demographic and pertinent social determinant information from each participant at the beginning of each focus group. All other data is generated from semi-structured focus groups and interviews. Notetakers either handwrote or typed notes to be used later for coding and analysis. Additionally, notes were taken on large Flipboards for all participants to see during each focus group.

Analysis of social determinant data as well as some focus group data was performed using Python 2.4.7 to generate descriptive statistics and accompanying graphics. Focus group notes were hand-coded to identify overarching themes and to organize cultural insights provided form participants in each group. Large lists, such as common foods, were analyzed using word clouds to associate common items with greater weights. The results presented below are stacked by Caribbean island where appropriate. Otherwise, the data are presented as a whole with consideration of all groups.

<u>Social Determinants of Health Overview</u>

Age

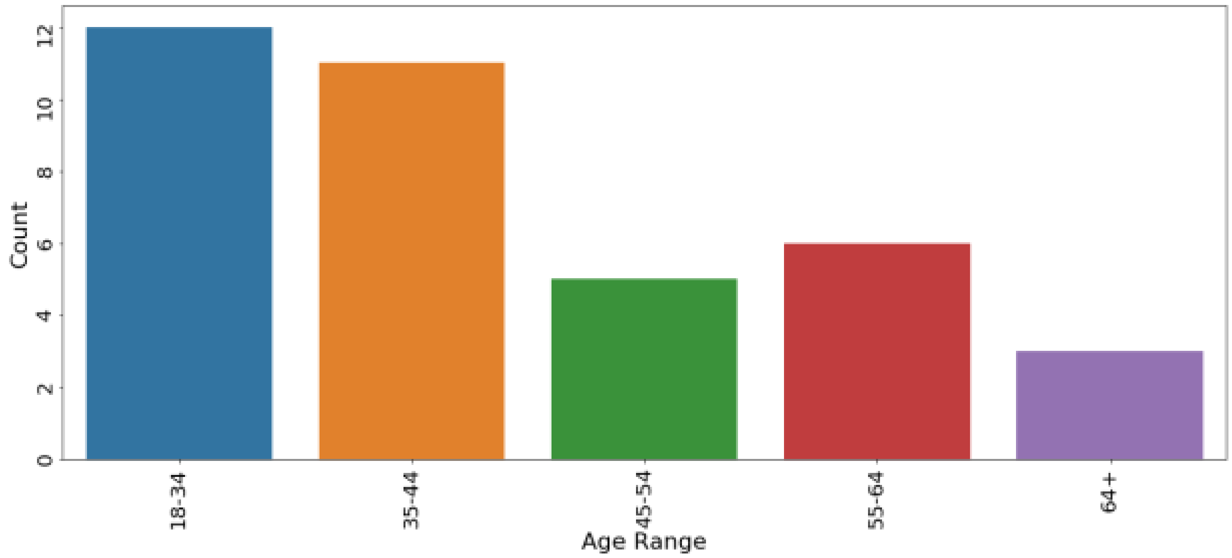

Participants of all focus groups averaged nearly 44 years old and ranged from 22 to 68 years old.

Gender

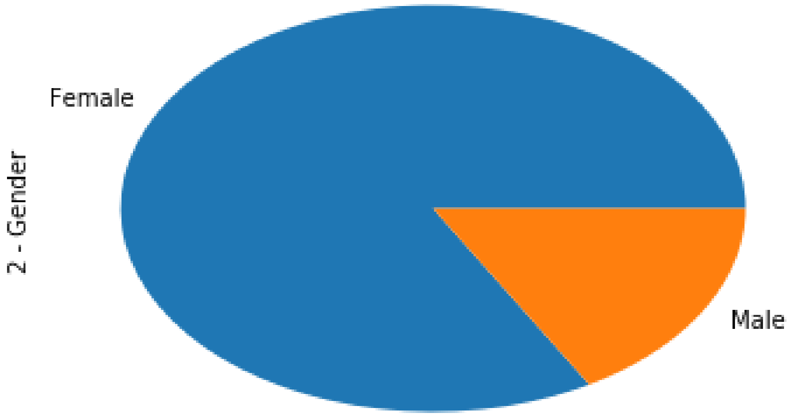

83.3% of participants are female and 16.7% participants are male.

Race

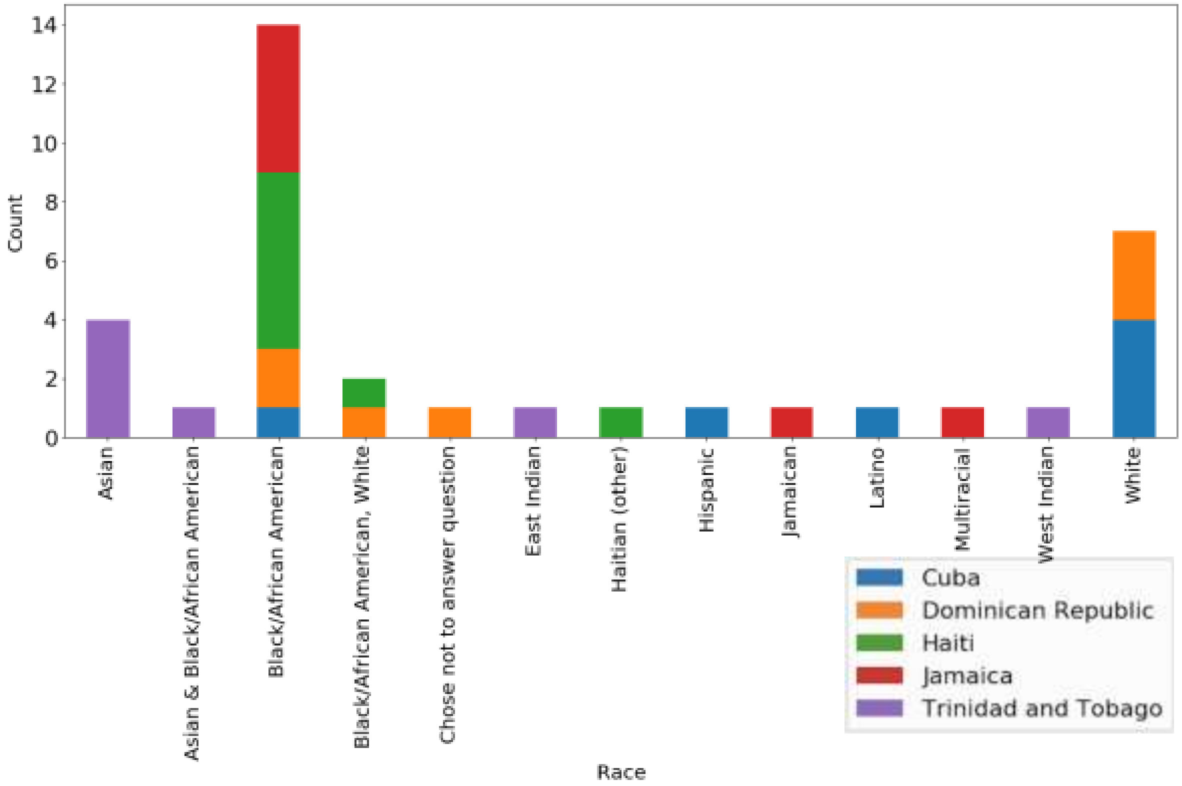

When participants were asked to select their race, their responses varied. Many selected the option for "Other" and provided the term they most identified with. Many participants expressed that they are often unsure what to select in these instances.

Education

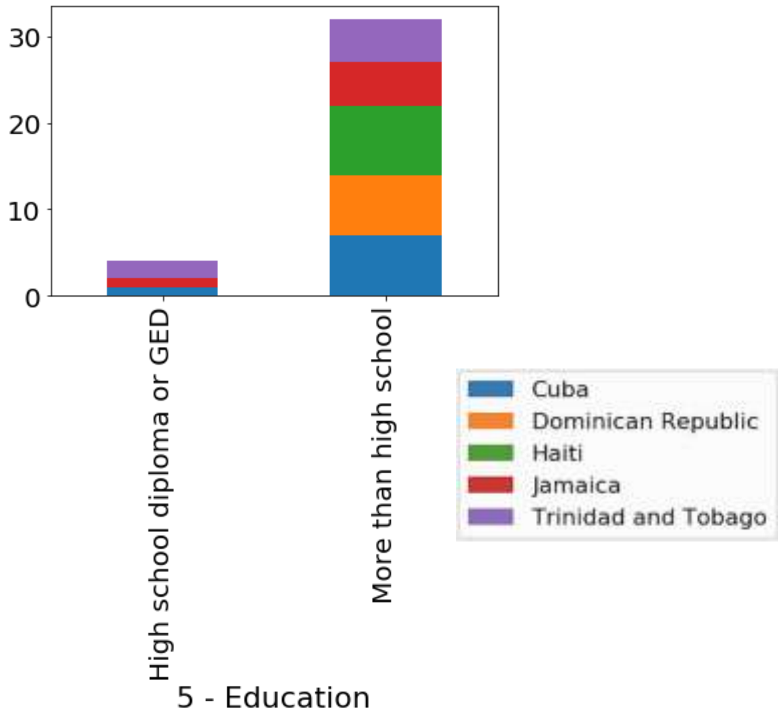

Most participants in all focus groups have more than a high school education.

Insurance

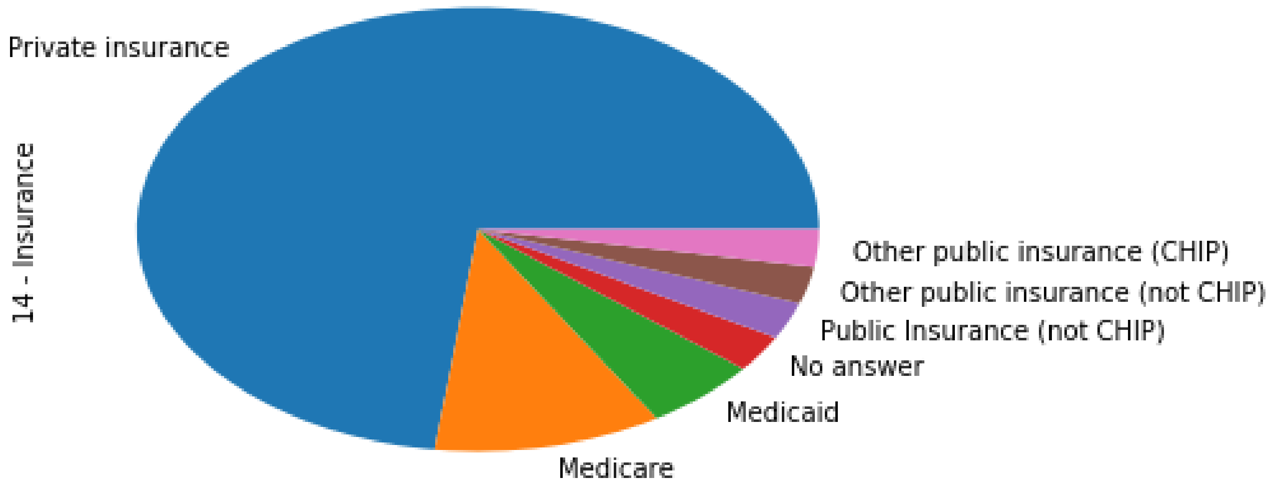

Most participants have private insurance (72.9%), followed by those with Medicare (10.8%).

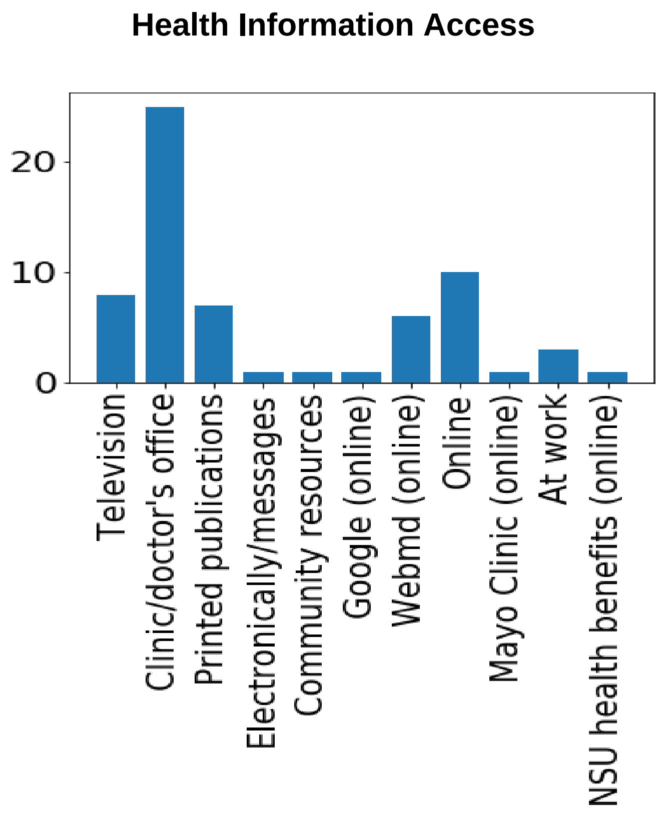

Most participants (39.1%) report that they primarily access health information at a clinic or doctor's office. A total of 28.7% of participants access health information online (via Google, WebMD, Mayo Clinic, or NSU Health Benefits online, or another website).

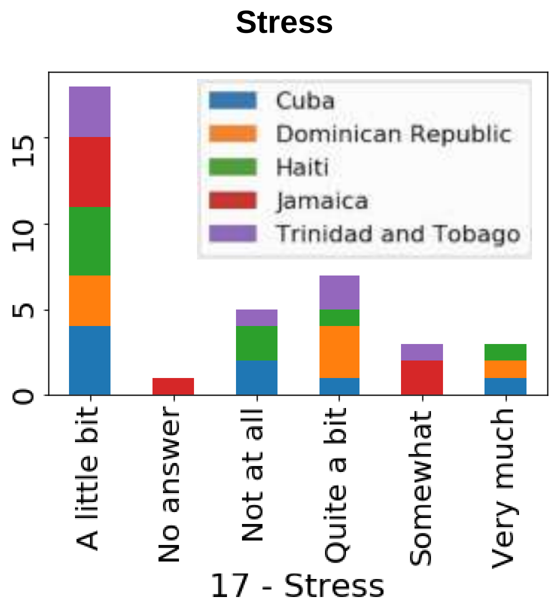

While 48.6% of participants report feeling "a little bit stressed," 18.9% report feeling "quite a bit" stressed.

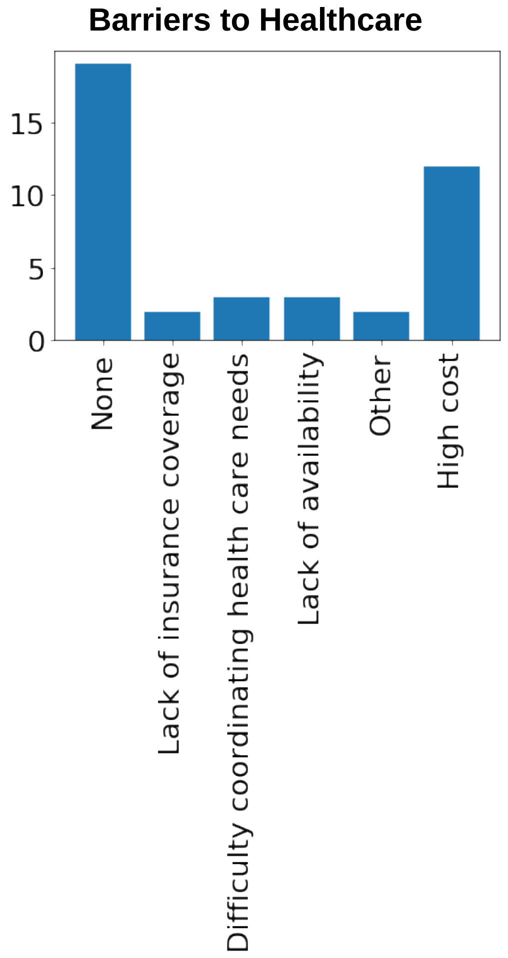

While most participants (46.3%) face no barriers accessing healthcare, over half of participants face at least one barrier. The most common barrier is the high cost of health care (expressed by 29.3% of participants).

Focus Group Results – Material Selections

For every packet of material, participants were asked to vote for the page(s) they thought were most useful and/or the pages they thought were best presented. The results are displayed below.

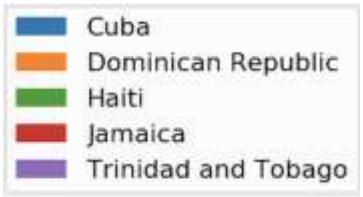

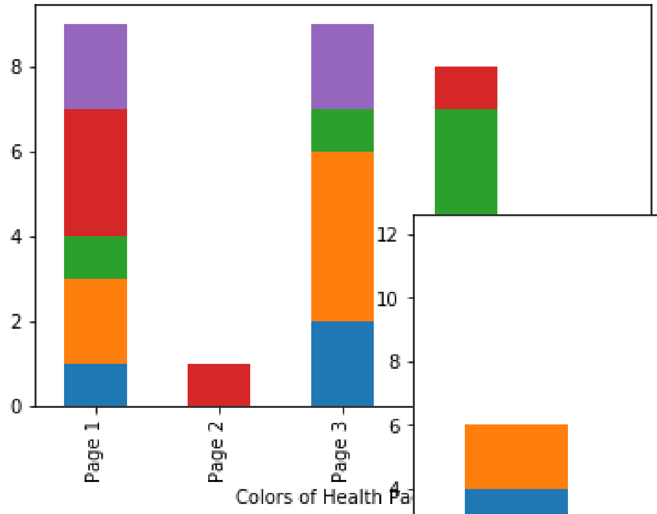

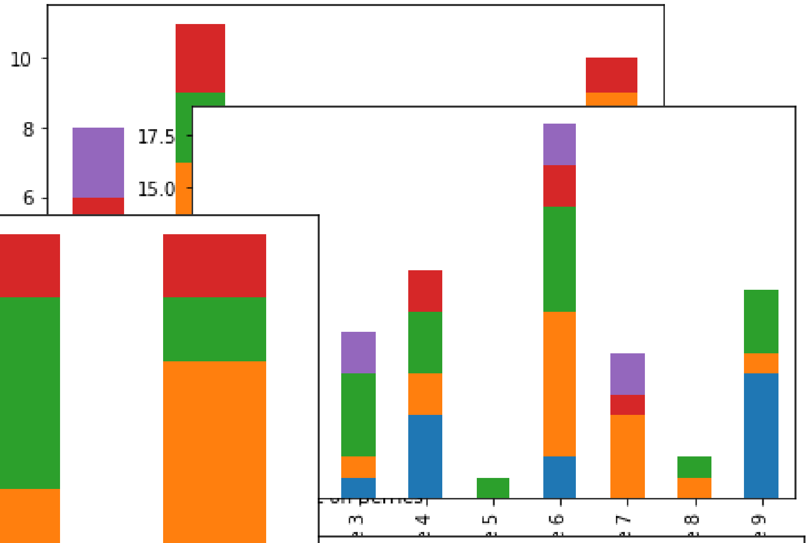

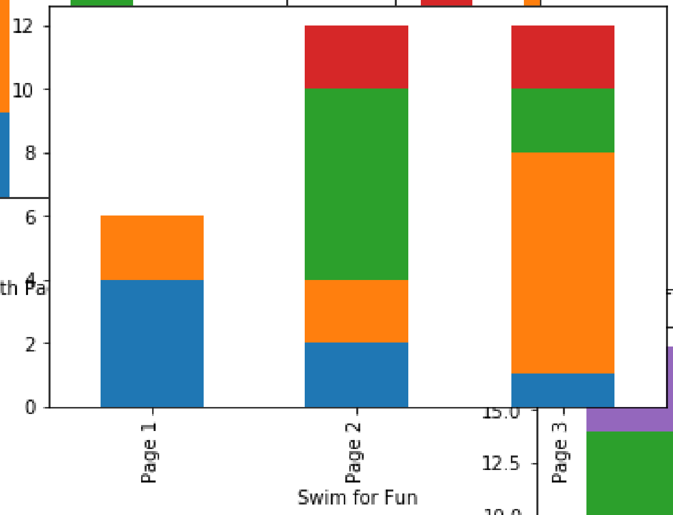

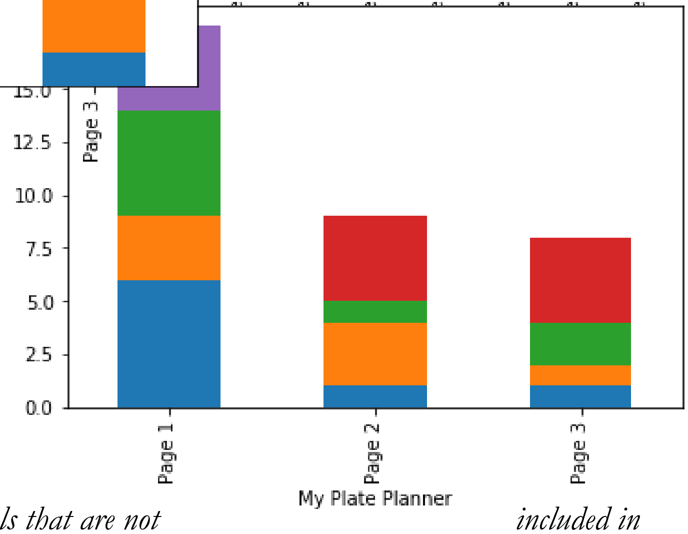

Common Food Items and Meals

Throughout each focus group, participants mentioned food items and meals that are not included in present nutritional material. Additionally, participants provided insight as to food items and meals that are common in their respective cultures to be included in future material tailored to Caribbean culture. The word cloud below provides a graphic that represents each food item or meal mentioned throughout all focus groups with larger items representing more common food items and meals.

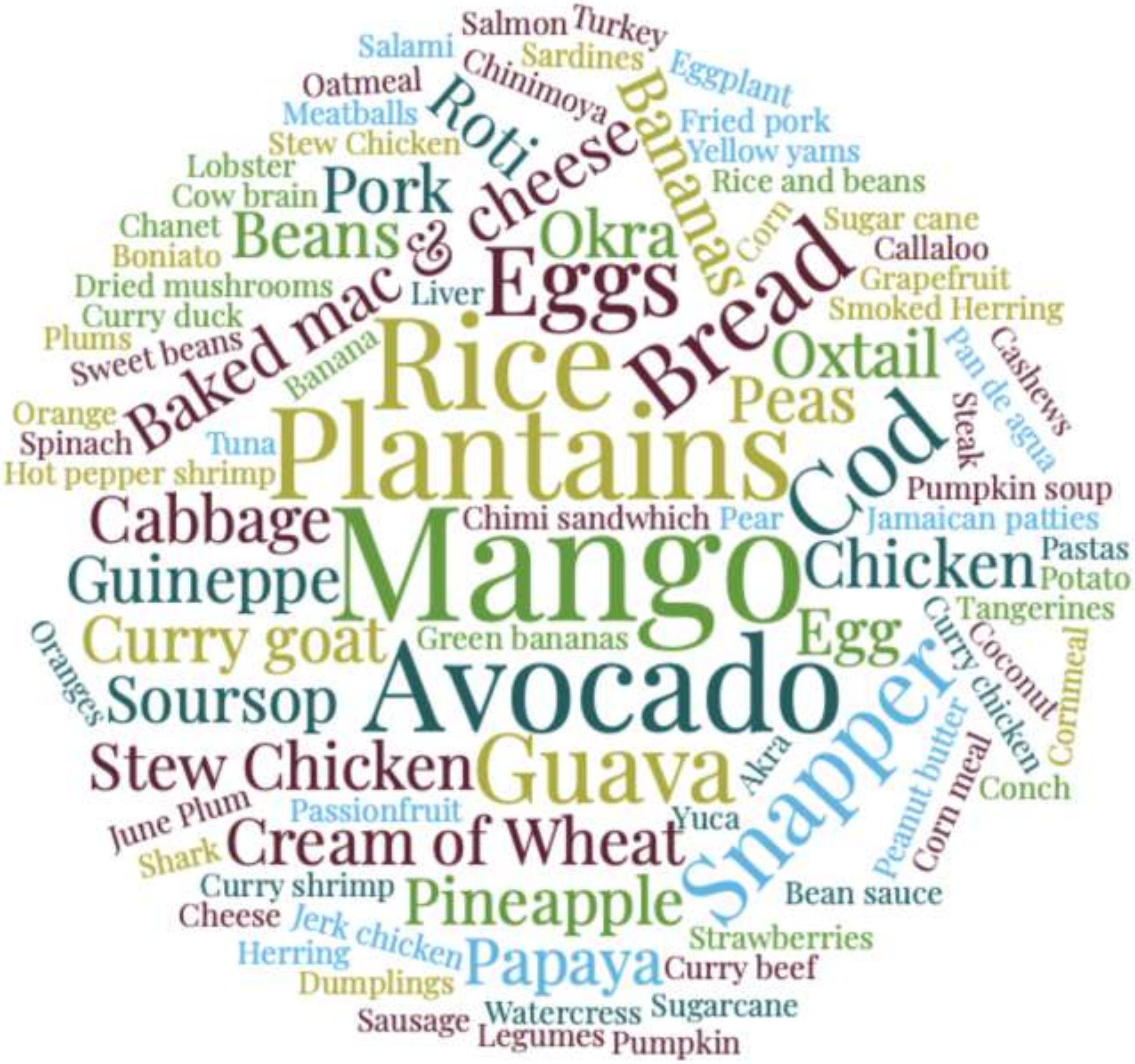
Salmon
Turkey
Salami
Sardines
Chinimoya
Eggplant
Oatmeal
Meatballs
Roti
Bananas
Fried pork
Stew Chicken
Yellow yams
Lobster
Cow brain
Pork
Baked mac & cheese
Rice and beans
Chanet
Beans
Okra
Corn
Sugar cane
Boniato
Callaloo
Dried mushrooms
Liver
Eggs
Bread
Grapefruit
Curry duck
Smoked Herring
Plums
Sweet beans
Banana
Rice
Oxtail
Pan de agua
Cashews
Orange
Peas
Steak
Spinach
Tuna
Plantains
Cod
Hot pepper shrimp
Pumpkin soup
Cabbage
Chimi sandwhich
Pear
Jamaican patties
Guineppe
Mango
Chicken
Pastas
Potato
Tangerines
Curry goat
Green bananas
Egg
Curry chicken
Coconut
Cornmeal
Oranges
Soursop
Avocado
Snapper
Peanut butter
Corn meal
Stew Chicken
Guava
Akra
Conch
June Plum
Passionfruit
Yuca
Shark
Cream of Wheat
Curry shrimp
Pineapple
Bean sauce
Cheese
Jerk chicken
Strawberries
Herring
Papaya
Curry beef
Dumplings
Sausage
Watercress
Sugarcane
Legumes
Pumpkin

Overlapping Food and Exercise Themes

The following table represents the most common food and drink items as well as most common exercise themes across all islands organized by type.

Fruits	• Mango<br>• Guava<br>• Avocado<br>• Papaya<br>• Soursop<br>• Pineapple
Meat and Fish	• Chicken (stew, curry)<br>• Goat<br>• Oxtail<br>• Pork<br>• Salami<br>• Snapper<br>• Cod<br>• Eggs<br>• Shrimp
Vegetables and Beans	• Cabbage<br>• Plantains<br>• Corn<br>• Pumpkin (butternut squash)<br>• Kidney Beans<br>• Beans<br>• Pigeon peas
Wheat and Grains	• Bread<br>• Roti<br>• Cream of Wheat<br>• Baked mac & cheese<br>• Rice (Jasmine, brown)
Drinks	• Natural, fresh fruit juice (with added milk/condensed milk and brown sugar)<br>• Coffee (sometimes black, sometimes with cream and sugar)<br>• Colas respective to island (i.e. Merengue Country Club Soda in the D.R.)<br>• Pepsi, Coke, Orange Fanta<br>• Malta
Exercise	• American football and swimming in pools are unrelatable and/or unpopular for exercise<br>• Dancing, baseball, cricket, and soccer are common for exercise and for entertainment<br>• Walking (in groups, with families, outdoors) is favored by all groups for exercise

Restaurants, Community Centers, and Grocery Stores

Each group provided lists of restaurants, community centers (including churches and places of worship), and grocery stores that are most commonly frequented. The word clouds below represent the most common sites with larger items representing the most common sites mentioned by participants.

Restaurants

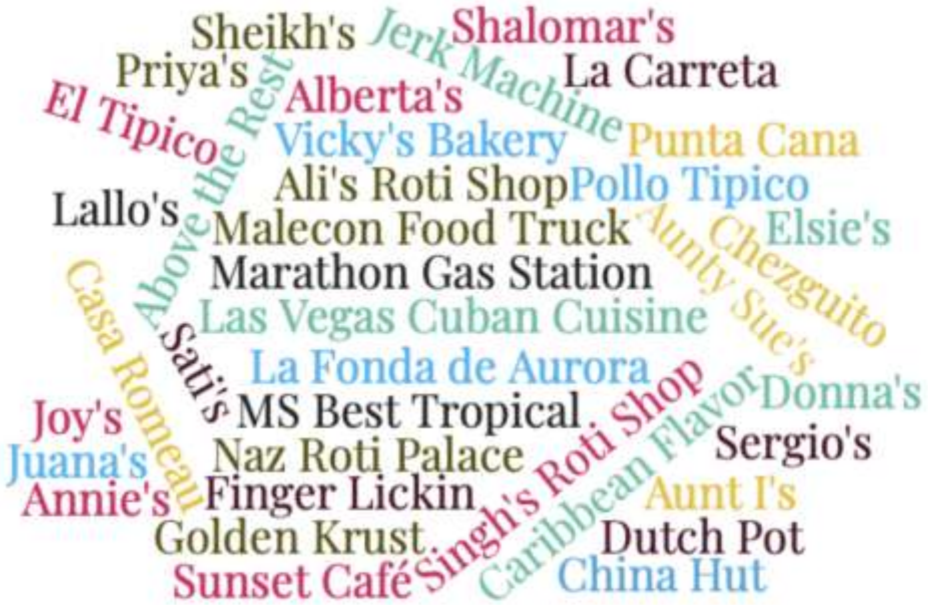

Grocery Stores

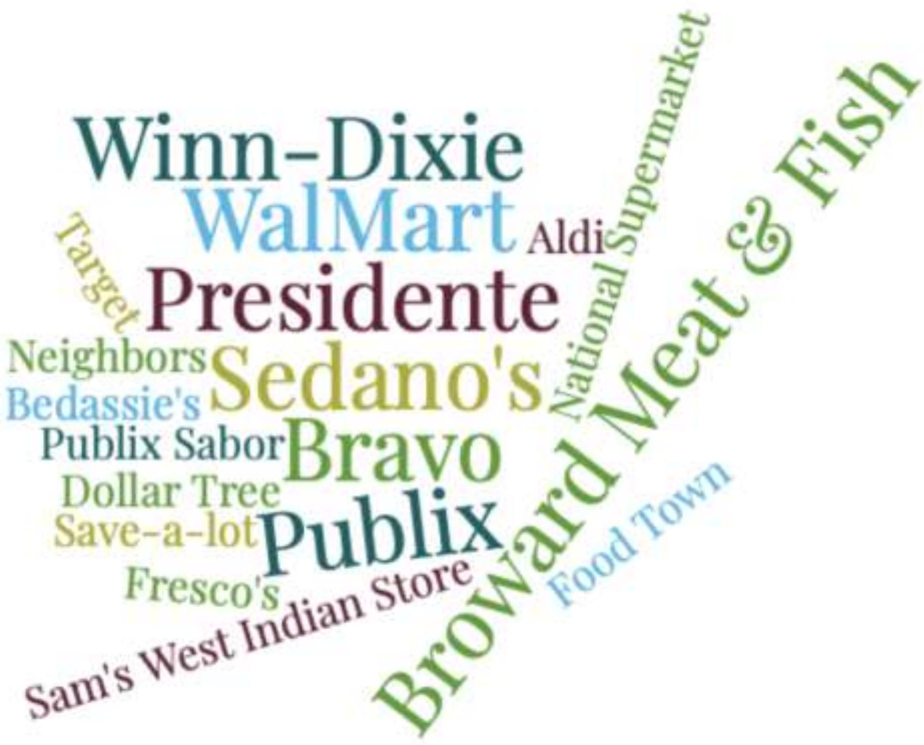

Community Centers

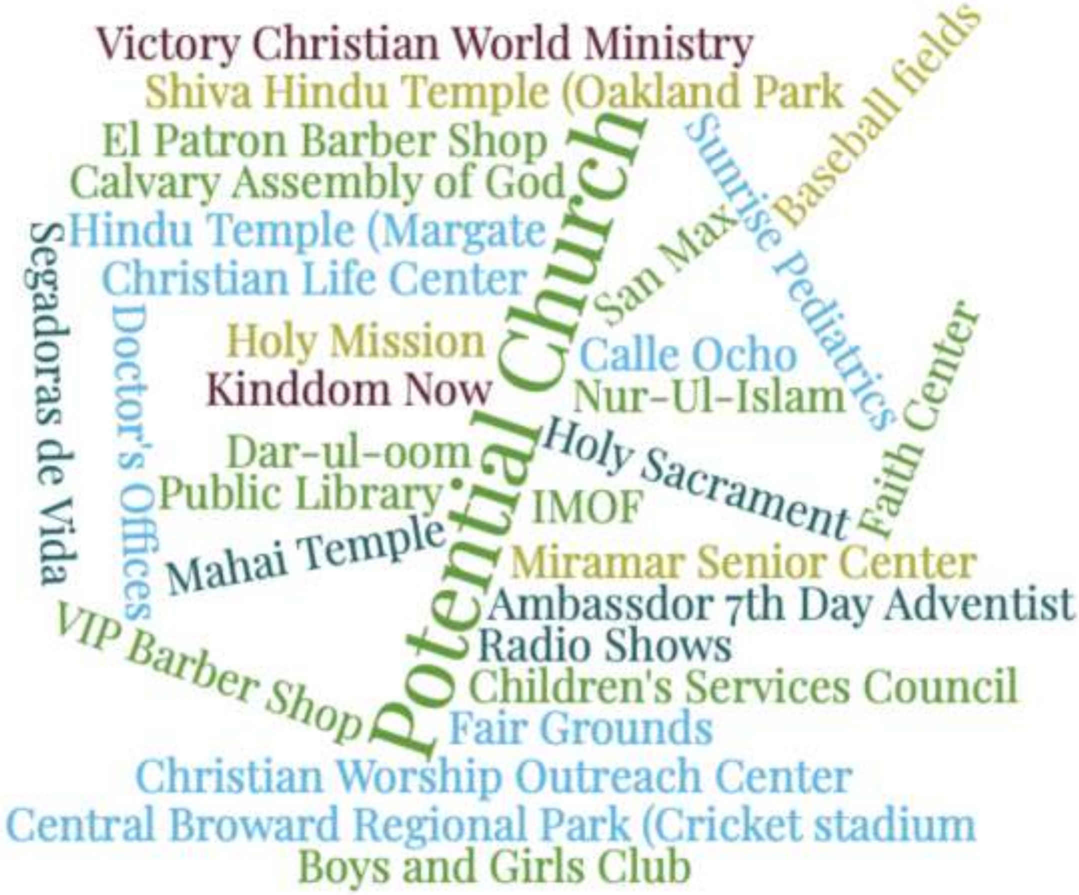

<u>Themes</u>

The following is a grouping of common themes resulting from all focus groups from each of the five Caribbean islands represented in this study.

Organization	o Colors: Participants value bright colors and color-coded information<br>o Layout: Bulleted information is easier to understand and more attractive; cluttered information is useless and can be easily ignored; logos repeatedly reduce visual fluidity<br>o Phrases: Key terms should be bolded and catchy slogans/phrases are favorable to participants
Information	o Exclude overused material (apple equals health, food pyramid, etc.)<br>o Calorie counts and nutritional information are highly valued<br>o Food equations (ex. 1 donut hole = 27 grapes) should be comparable (snacks compared to snacks, meals compared to meals, etc.)<br>o Include culturally appropriate exercises<br>▪ Swimming among African American women is uncommon<br>▪ Walking is favored by all groups and is extremely popular
Illustrations, Pictures, and Graphics	o Participants from all groups favor depictions of families (exercising) and happy children<br>o Crowded graphics are disliked (ex: My Plate Planner)<br>o Graphics should be clear and easy to understand (Page 2 of My Plate Planner was often mistaken for sign language)<br>o Donut holes (Colors of Health packet), candy bar (Soda? Think Again packet), and soda cans (Soda? Think Again packet) are not easily recognizable to many participants<br>o Inclusion of cultural items (dominos, wooden spoons) is encouraging and more representative<br>o Some items (i.e. hockey pucks) are uncommon in Caribbean culture
Cultural Insights	o Eggs are common in Caribbean cuisine, but nutritional value of eggs is often misinterpreted. Many believe that eggs are bad for you, so nutritional information about eggs is useful.<br>o Almonds aren't popular – peanuts are more popular<br>o **Portion size/control isn't popular or widely understood**<br>o **Measuring/following recipes when cooking isn't popular**<br>o Juicing is popular, but milk and sugar are often added<br>o Canned food isn't considered healthy<br>o **Consider using more culturally appropriate foods for comparison and inclusion**<br>o Colors of Health Packet – blueberries can be equated to sickness/medicine and (negatively received)<br>o Sports and exercise: baseball, cricket, dancing, and soccer are more popular than American football<br>o Some cultures commonly indulge in spicy foods (especially use of scotch bonnet peppers) and others are impartial
